# Proteasome inhibitors act as bifunctional antagonists of human immunodeficiency virus type 1 latency and replication

**DOI:** 10.1186/1742-4690-10-120

**Published:** 2013-10-24

**Authors:** Leia K Miller, Yoshifumi Kobayashi, Chiann-Chyi Chen, Timothy A Russnak, Yacov Ron, Joseph P Dougherty

**Affiliations:** 1 Department of Molecular Pharmacology, Rutgers, The State University of New Jersey - Robert Wood Johnson Medical School, 675 Hoes Lane, Piscataway, NJ 08854, USA; 2 Microbiology and Molecular Genetics Graduate Program, Graduate School of Biomedical Sciences, Rutgers, The State University of New Jersey, New Brunswick, NJ, USA

**Keywords:** HIV-1, Viral latency, Viral replication, Proteasome inhibitors, Antiviral, Bifunctional

## Abstract

**Background:**

Existing highly active antiretroviral therapy (HAART) effectively controls viral replication in human immunodeficiency virus type 1 (HIV-1) infected individuals but cannot completely eradicate the infection, at least in part due to the persistence of latently infected cells. One strategy that is being actively pursued to eliminate the latent aspect of HIV-1 infection involves therapies combining latency antagonists with HAART. However, discordant pharmacokinetics between these types of drugs can potentially create sites of active viral replication within certain tissues that might be impervious to HAART.

**Results:**

A preliminary reverse genetic screen indicated that the proteasome might be involved in the maintenance of the latent state. This prompted testing to determine the effects of proteasome inhibitors (PIs) on latently infected cells. Experiments demonstrated that PIs effectively activated latent HIV-1 in several model systems, including primary T cell models, thereby defining PIs as a new class of HIV-1 latency antagonists. Expanding upon experiments from previous reports, it was also confirmed that PIs inhibit viral replication. Moreover, it was possible to show that PIs act as bifunctional antagonists of HIV-1. The data indicate that PIs activate latent provirus and subsequently decrease viral titers and promote the production of defective virions from activated cells.

**Conclusions:**

These results represent a proof-of-concept that bifunctional antagonists of HIV-1 can be developed and have the capacity to ensure precise tissue overlap of anti-latency and anti-replication functions, which is of significant importance in the consideration of future drug therapies aimed at viral clearance.

## Background

Human immunodeficiency virus type 1 (HIV-1) infection is presently incurable necessitating life-long drug treatment [1]. HIV-1 is able to persist within its cellular host by entering a reversible dormant state, termed latency, which provides protection from the immune system and antiviral pharmaceuticals. Consequently, latent HIV-1 is able to persist indefinitely awaiting activation, upon which it can reestablish a productive infection in the absence of highly active antiretroviral therapy (HAART) (Latency Reviewed in [2]).

Currently, one strategy to abolish latent infection involves treating patients with a latency antagonist concomitantly with HAART to prevent new infections and the reestablishment of the latent reservoir upon the activation of latent virus [2-8]. A major reservoir of latent infection *in vivo* is within memory CD4^+^ T cells, [9] although other cell types have been reported to harbor latent HIV-1, including cells of myeloid origin. Importantly, latently infected cells can be found in tissues that are resistant to effective penetration of at least some HAART drugs [10-17]. For instance, the brain was reported to house latently infected cells [10,17-21] yet the blood–brain barrier (BBB) can restrict the penetrance of some antiretroviral drugs into the brain [22-28]. In light of this, it may be important to not only treat patients with both latency activators and HAART simultaneously, but to ensure their concurrent delivery to the same tissue and cellular compartments.

The 26S proteasome is composed of two regulatory 19S subunits that abut a catalytic 20S core subunit and as a whole is responsible for the degradation of ubiquitinated proteins in the cell [29]. Interestingly, the proteasome is involved in promoting HIV-1 replication via its specific degradation of the APOBEC3 family of HIV-1 restriction factors in the presence of the viral protein Vif (Reviewed in [30,31]). Surprisingly, as delineated in this study, it was also found that the proteasome is involved in maintaining HIV-1 latency. The fact that the proteasome positively influences both HIV-1 replication and latency makes it a unique drug target whose inhibition has the potential to elicit dual antiviral effects. The development of a drug that exhibits bifunctional antagonism of both aspects of the viral life cycle would help to address concerns regarding the insufficient penetration of HAART into some tissues harboring latently infected cells.

In this report, evidence that proteasome inhibitors (PIs) hinder both HIV-1 latency and replication is presented. Here, it is shown that PIs activate latent HIV-1 in several *in vitro* model systems, including two primary human CD4^+^ T cell model systems. Consequently, PIs represent a new class of HIV-1 latency antagonists. Additionally, this study confirms that PIs inhibit HIV-1 infectivity. Finally, it is demonstrated that PIs antagonize both HIV-1 latency and replication in a sequential manner in virus-producing cells. These results introduce a novel proof-of-concept that effective bifunctional HIV-1 antagonists can be developed.

## Results

### PIs activate latent HIV-1 transcription, gene expression, and virus production

**Figure 1:**
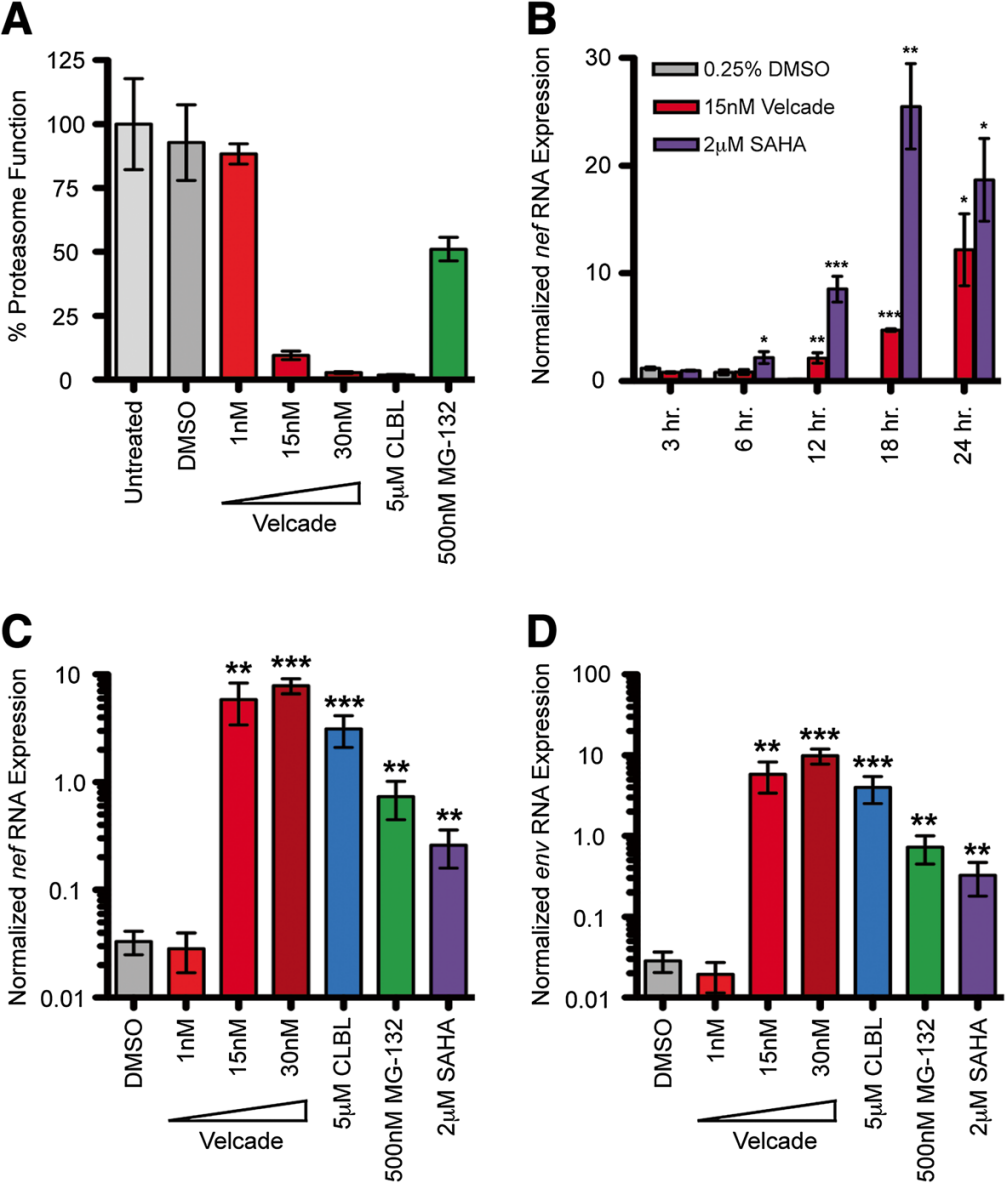
**PIs activate latent HIV-1 transcription. A**. OM-10.1 cells were treated with PIs as indicated and proteasome activity was assessed two hours post-treatment. The values shown specify the percent proteasome function compared to untreated cells, whose function was set to 100%. **B**. OM-10.1 cells were treated with 15nM Velcade for the indicated time course. At each time point, *nef* RNA levels were analyzed via reverse transcription-quantitative PCR. RNA expression values were calculated via ΔΔC(t) method with the values normalized to the expression level of GAPDH in each sample. **C**. OM-10.1 cells were treated with PIs as indicated and *nef* RNA and **D**. *env* RNA levels were analyzed 72 hours post-treatment via reverse transcription-quantitative PCR (values calculated as above). Error bars indicate SEM. Asterisks indicate a significant difference (* p<0.05; ** p<0.01; *** p<0.001) in RNA expression levels between drug-treated cells and DMSO-treated (negative control) cells. P-values calculated using one-tailed Student’s t test. Cells were treated with SAHA as a positive control. Velcade was used in three different concentrations where indicated to illustrate concentration dependence. The figure represents average values from three independent experiments.

A preliminary reverse genetic screen in a HeLa cell model of HIV-1 latency implicated the 26S proteasome as a novel cellular regulator of the maintenance of HIV-1 latency (unpublished data). As the involvement of the proteasome in the maintenance of latency was unexpected, we chose to further validate its role through the use of PIs. Latently infected cells were treated with PIs to analyze the activation of proviral transcription. OM-10.1 cells, which are a clonal population of HL-60 promyelocytes that are latently infected with the replication-competent HIV-1_LAV_ strain [32-36], were treated with the PI Velcade. Velcade is an inhibitor of the chymotrypsin-like activity of the 20S proteasome core particle [37,38] and is also FDA approved for the treatment of multiple myelomas, leukemias, and lymphomas [37,39-42]. Velcade inhibited proteasome function within two hours (Figure 1A), and resulted in a significant increase in the level of *nef*-containing viral RNAs in OM-10.1 cells as early as 12 hours post-treatment (Figure 1B). To confirm the activation of latent viral transcription, two additional inhibitors of the chymotrypsin-like activity of the proteasome (clasto-Lactacystin β-lactone (CLBL) and MG-132 [37,38]) were used to assess the accumulation of both *nef*- and *env*-containing viral RNAs. CLBL and MG-132 also inhibited proteasome function within two hours (Figure 1A) and significantly induced the expression of viral RNAs (Figure 1C and D). Of note, concentration dependence is shown for Velcade due to its clinical relevance. Otherwise, optimal PI concentrations were selected and utilized based on knowledge of IC_50_ concentrations as well as cytotoxicity and dose response profiles (data not shown).

**Figure 2:**
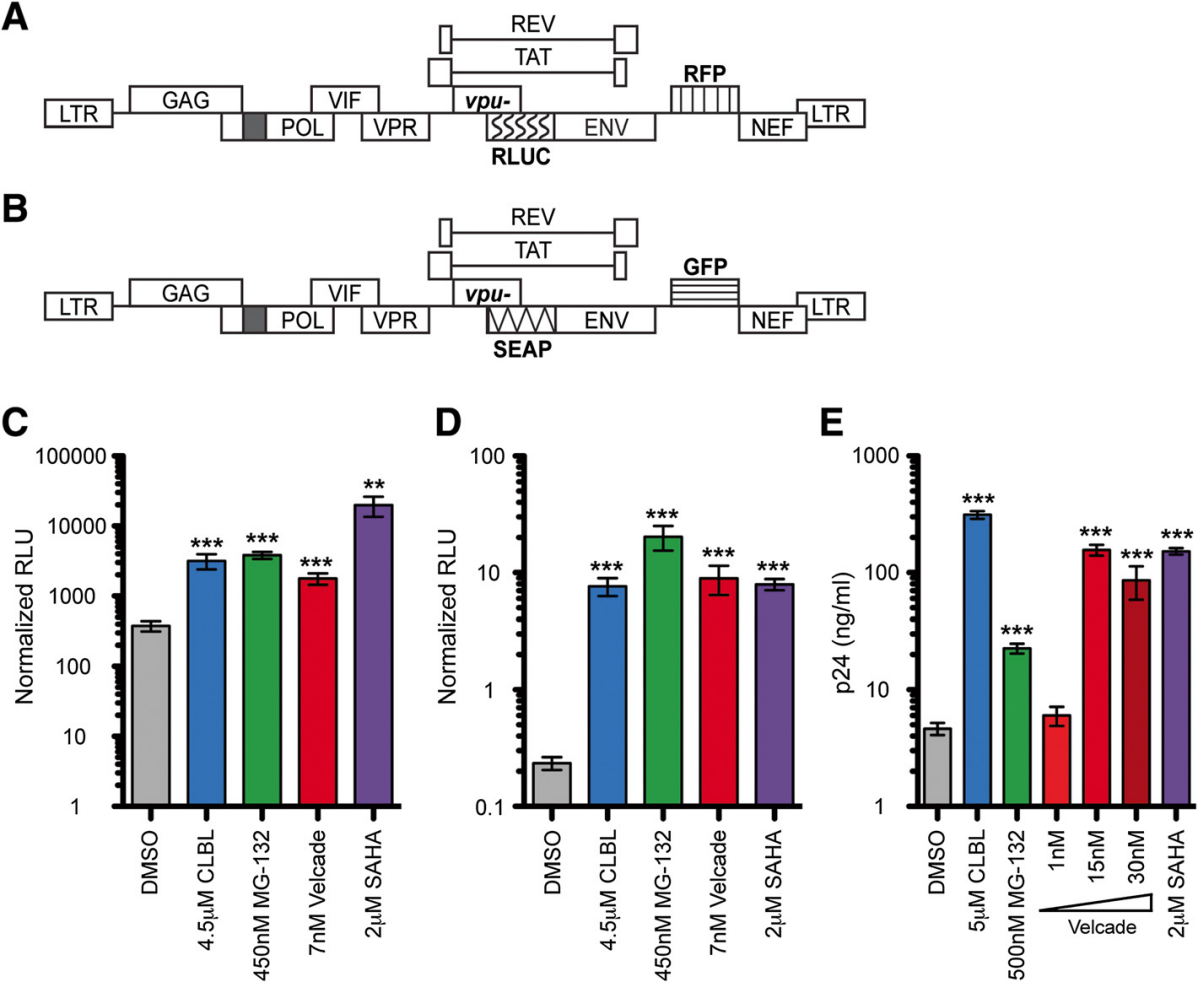
**PIs activate latent HIV-1 gene expression in tissue culture model systems. A**. Schematic of the RLUC/RFP construct used to establish latently infected HeLa#14 cells. The *vpu* gene start codon is mutated for robust reporter gene expression. The black box represents a deletion and the patterned boxes represent RLUC and RFP reporter gene insertions. Together, the deletion in *pol* and the insertion in *env* render the vector replication-incompetent. **B**. Schematic of the SEAP/GFP construct used to establish latently infected 24ST1NLESG cells. The *vpu* gene start codon is mutated for robust reporter gene expression. The black box represents a deletion and the patterned boxes represent SEAP and GFP reporter gene insertions. Together, the deletion in *pol* and the insertion in *env* render the vector replication-incompetent. **C**. HeLa#14 cells were treated for 48 hours with PIs as indicated and RLUC activity was measured. The values shown indicate RLUs normalized to protein concentrations. **D**. 24ST1NLESG cells were treated with CLBL for 48 hours and MG-132 and Velcade for 72 hours at the indicated concentrations, and SEAP activity was analyzed. The values shown indicate RLUs normalized to the number of live cells. **E**. OM-10.1 cells were treated for 72 hours with PIs as indicated and p24 levels in the supernatant were analyzed via p24 ELISA. p24 levels (ng/mL) were calculated using standard curve values. Error bars indicate SEM. Asterisks indicate significant differences (** p<0.01; *** p<0.001) between drug-treated cells and DMSO-treated (negative control) cells. P-values were calculated using one-tailed Student’s t test. Cells treated with SAHA served as positive controls. The figure represents average values from three independent experiments.

Next, OM-10.1 cells and additional tissue culture-based latency model systems (HeLa#14 cells [43] and 24ST1NLESG cells [44]) were treated with PIs to analyze the activation of proviral gene expression and virus production. HeLa#14 cells are a clonal population of HeLa cells that are latently infected with an HIV-1_NL4-3_-based reporter construct RLUC/RFP. Briefly, the vector is rendered replication-incompetent via deletions in *pol* and *env* while red fluorescence protein (RFP) is expressed as an early gene product from the *nef* position and *Renilla* luciferase (RLUC) is expressed as a late gene product from the *env* position (Figure 2A) [43]. 24ST1NLESG cells are a clonal population of SupT1 cells, a human CD4^+^ T cell line, latently infected with an HIV-1_NL4-3_-based reporter construct SEAP/GFP. Briefly, the vector is rendered replication-incompetent via deletions in *pol* and *env* while green fluorescence protein (GFP) is expressed as an early gene product from the *nef* position and secreted alkaline phosphatase (SEAP) is expressed as a late gene product from the *env* position (Figure 2B) [44]. All three PIs were able to significantly induce HeLa#14 cell RLUC expression (Figure 2C), 24ST1NLESG cell SEAP expression (Figure 2D), as well as induce viral particle production from OM-10.1 cells, as evidenced by a significant increase in HIV-1 capsid protein (p24) concentration in the supernatant (Figure 2E). Again, PI concentrations and treatment durations were selected and utilized based on knowledge of IC_50_ concentrations as well as cytotoxicity and dose response profiles (data not shown). It should be noted that although the expression of RLUC in HeLa#14 cells treated with DMSO appears to be high, it does not indicate that DMSO activates latent HIV-1 gene expression, as the magnitude of RLUC expression is equivalent in untreated HeLa#14 cells cultured for 48 hours in a small-well format (data not shown). Despite this relatively high background, PIs significantly induce RLUC expression in these cells (greater than 5-fold) over DMSO.

**Figure 3:**
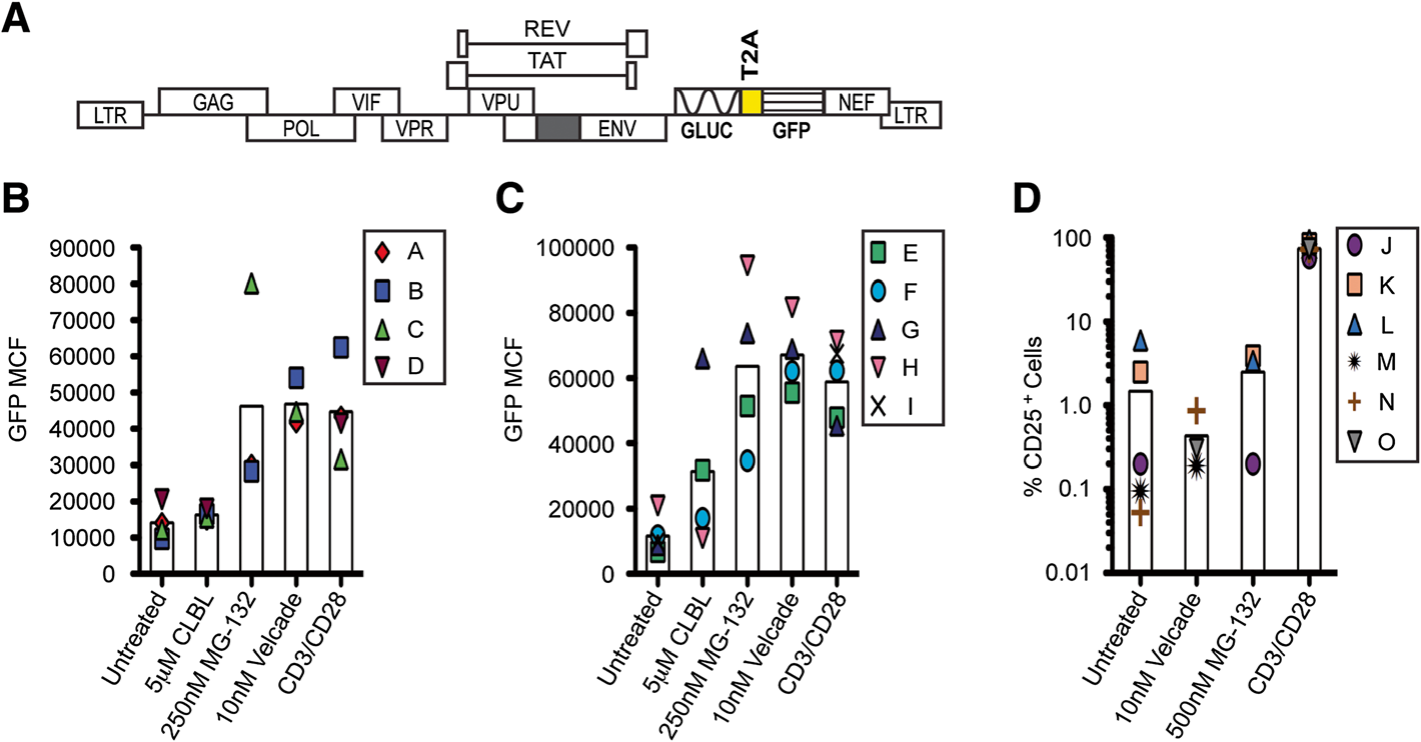
**PIs activate latent HIV-1 gene expression in primary CD4**^**+ **^**T cell models. A**. Schematic of gGnΔ construct used to establish latently infected T_CM_-like and *BCL2*-transduced cells. Black box represents a deletion in *env*, which renders this construct replication-incompetent. Patterned boxes indicate GLUC and GFP reporter gene insertions. The yellow box specifies a T2A sequence, which directs bicistronic expression [88,89]. **B**. T_CM_-like cells and **C**. *BCL2*-transduced primary CD4^+^ T cells were treated for 48 hours with PIs as indicated. Flow cytometry was performed and the values shown indicate GFP mean channel fluorescence (MCF). All cells treated with PIs were simultaneously treated with Raltegravir to prevent the integration of as yet unintegrated viral genomes. MG-132 and Velcade treatments significantly activated latent virus in both T_CM_-like cells (p<0.05; p<0.001, respectively) and in *BCL2*-transduced cells (p<0.01; p<0.001, respectively) in comparison to untreated (negative control) cells. P-values calculated using one-tailed Student’s t-test. **D**. Resting CD4^+^ T cells isolated from healthy donor PBMCs were treated with 10 nM Velcade or 500 nM MG-132 for 48 hours and then incubated with FITC-conjugated CD25 antibody. The percentage of CD25^+^ cells in each sample was determined via flow cytometry. In all experiments, the vertical open bars represent average values from all healthy donors while symbols represent individual donor results; each donor result represents a singlicate experiment. Cells treated with CD3/CD28 activating beads served as positive controls.

The effects of proteasome inhibition on proviral gene expression in two latent HIV-1 primary human CD4^+^ T cell models were also studied. To start, HIV-1 virions were produced using a replication-incompetent HIV-1_NL4-3_-based reporter construct gGnΔ in which *Gaussia* luciferase (GLUC) and GFP are expressed as early gene products from the *nef* position (Figure 3A). This reporter virus was then used to establish a latent HIV-1 infection in primary human CD4^+^ T cells, isolated from peripheral blood mononuclear cells (PBMCs) collected from healthy donors, via two distinct methods. The first involved the infection of non-polar CD4^+^ T cells, which are considered to be the *in vitro* counterparts of latently infected central memory T cells *in vivo*[45]. Naïve human CD4^+^ T cells were isolated, activated in a non-polarizing environment, infected with the gGnΔ virus, and then cultured for seven days to establish a latent infection. These cells are referred to as T_CM_-like cells [45]. The second method involved the use of primary human CD4^+^ T cells transduced with a *BCL2* expression vector, which mimic both central memory and effector memory T cells [46]. The *BCL2*-transduced cells were activated, infected with the gGnΔ virus, and cultured for seven days to permit the establishment of viral latency [46]. The latently infected T_CM_-like primary and *BCL2*-transduced human CD4^+^ T cells were treated with PIs and analyzed for latent viral gene expression 48 hours later. Despite the expected donor-to-donor variability, MG-132 and Velcade treatments significantly induced the expression of GFP (Figure 3B and C) in both latent, primary human CD4^+^ T cell models. It should be noted that CLBL caused significant cytoxicity in both of these primary cell model systems, which made it difficult to assess the latency antagonist effect of CLBL here. However, the cytotoxic effects of CLBL were much less pronounced in the tissue culture model systems tested in which CLBL significantly activated latent virus. Overall, two of the three PIs clearly activated latent virus in the two primary cell models tested, and all three PIs activated latent virus in the three tissue culture model systems tested. These results strongly suggest that PIs act as latency antagonists.

We also examined the activation status of uninfected primary human resting CD4^+^ T cells following exposure to MG-132 and Velcade. Resting human CD4^+^ T cells cultured in the presence of Velcade or MG-132 for 48 hours did not become activated, as evidenced by a lack of expression of the T cell activation marker CD25 (Figure 3D). These results are in concert with previous studies, which indicated that PIs do not activate primary human T cells [47,48].

### PIs decrease viral titers and inhibit HIV-1 infectivity

**Figure 4:**
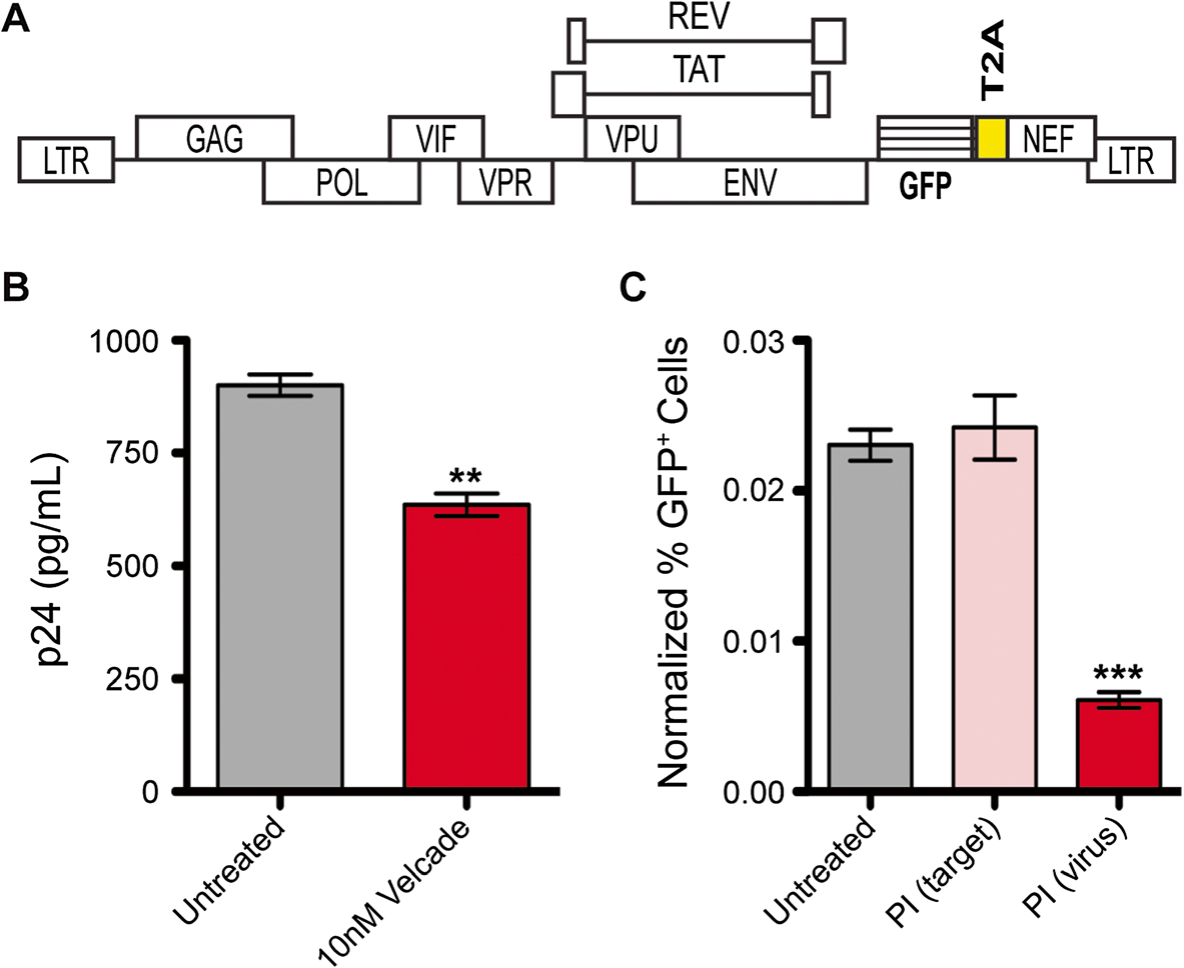
**Primary CD4**^**+ **^**T cells treated with Velcade produce fewer, less infectious virions. A**. Schematic of Gn construct used to produce replication-competent virus for this experiment. The patterned box indicates GFP reporter gene insertion and the yellow box specifies a T2A sequence, which directs bicistronic expression [88,89]. **B**. CD4^+^ T cells isolated from healthy donor PBMCs were activated and infected with Gn virus. Six hours post-infection, cells were either treated with 10 nM Velcade or left untreated as a positive control. Seventy-two hours post-infection, virus-containing supernatants were collected and p24 levels were analyzed via p24 ELISA. Values shown represent p24 levels (pg/mL) calculated using standard curve values. **C**. Untreated and Velcade-treated virus containing supernatants were used to infect HeLaT4 cells. Untreated supernatants were also used to infect HeLaT4 cells in the presence of 10nM Velcade [PI (target)] to control for effects that may arise from residual Velcade in the inoculum collected from Velcade-treated virus producing cells [PI (virus)]. Forty-eight hours post-infection, GFP^+^ cell numbers were analyzed via flow cytometry. Values shown indicate percent GFP^+^ cells normalized to the p24 (ng/ml) concentration of the inoculating viral supernatant. Error bars indicate SEM. Asterisks indicate significant differences (** p<0.01; *** p<0.001) between Velcade treatments and untreated (positive control) cells. P-values calculated using one-tailed Student’s t test. The figure represents average values from three independent experiments, each of which utilized primary cells isolated from different healthy donors.

Previous reports indicate that proteasomal inhibition in producer cells decreases HIV-1 titers and virion infectivity [49-53]. To confirm the findings, virions were generated via transfection of the replication-competent HIV-1_NL4-3_-based reporter construct Gn, in which GFP is expressed as an early gene product from the *nef* position, (Figure 4A) into HEK293T cells. The reporter virus was used to infect activated primary human CD4^+^ T cells isolated from PBMCs collected from healthy donors. Six hours post-infection, cells were either treated with 10 nM Velcade or left untreated for 72 hours. The resultant virus-containing supernatants were collected and p24 concentrations were measured. As shown in Figure 4B, supernatant collected from untreated virus-producing cells contained approximately 30% more p24 than supernatant collected from Velcade-treated virus-producing cells indicating that PIs reduce viral output. To analyze infectivity, the viral supernatants were used to infect HeLaT4 cells. As a control for potential effects on viral infectivity arising from residual PI in the inoculums collected from PI-treated virus-producing cells, 10 nM Velcade was added to some HeLaT4 cells as they were infected with inoculums collected from untreated virus-producing cells. Forty-eight hours post-infection, the number of infected (GFP^+^) HeLaT4 cells were counted and normalized to the p24 concentrations of inoculating viral supernatants. In a single round of infection, virus collected from infected, Velcade-treated primary CD4^+^ T cells [PI (virus)] was only able to infect approximately one fourth the number of HeLaT4 cells as virus collected from infected, untreated primary CD4^+^ T cells (Figure 4C). The addition of Velcade to HeLaT4 cells at the time of infection [PI (target)] did not have an effect on viral infectivity suggesting that residual PI in the inoculum collected from PI-treated virus-producing cells is not responsible for the reduction in infectivity observed. These results also suggest that PIs reduce viral output and virion infectivity when added to virus-producing cells but do not inhibit the infection of PI-treated target cells. This is in agreement with previous findings [51-54].

### PIs are bifunctional antagonists of HIV-1

**Figure 5:**
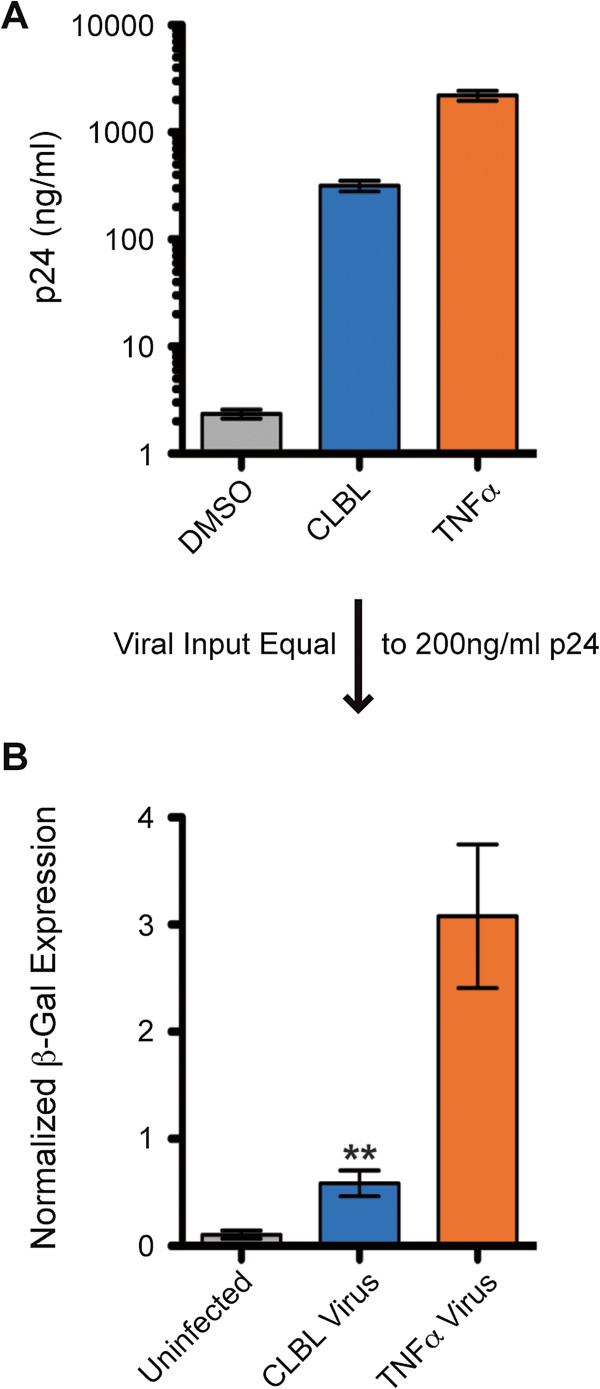
**HIV-1 virions produced via PI-mediated activation of latent virus exhibit reduced infectivity. A**. OM-10.1 cells were treated with 50 ng/ml TNFα to stimulate the production of positive control viral particles, with 5 μM CLBL to stimulate the production of viral particles under the influence of proteasome inhibition or with DMSO as a negative control. Seventy-two hours post-treatment, viral supernatants were collected and p24 levels were analyzed via p24 ELISA. Values shown represent p24 levels (ng/mL) calculated using standard curve values. **B**. CLBL and TNFα treated virus-containing supernatants were diluted to a p24 concentration of 200 ng/ml and used to infect U373-MAGI-CXCR4_CEM_ cells. Forty-eight hours post-infection, β-Gal expression was quantified using the β-Gal Enzyme Assay System (Promega, Madison, WI) and standard curve values. Numbers depict β-Gal expression normalized to protein concentrations. Error bars indicate SEM. Asterisks indicate a significant difference (p<0.01) in β-Gal expression between cells infected with TNFα treated virus and CLBL treated virus. P-value was calculated using one-tailed Student’s t test. Results represent average values from three independent experiments.

The results demonstrating that PIs can activate latent HIV-1, reduce viral output, and inhibit HIV-1 infectivity suggested the feasibility of antagonizing both latency and replication using a single pharmaceutical. To test this, OM-10.1 cells were treated with the PI CLBL to analyze the infectivity of virions following the activation of latent HIV-1. OM-10.1 cells were treated with either TNFα, to stimulate the production of positive control virus, or with CLBL, to stimulate the production of virus under the influence of proteasome inhibition (CLBL was chosen as it is the most potent activator of virus production in OM-10.1 cells and is therefore more comparable to TNFα than the other PIs). Seventy-two hours post-treatment, virus-containing supernatants were collected and p24 concentrations were measured. Figure 5A illustrates the magnitude of the induction of p24 production observed following the treatment of OM-10.1 cells with these two activators. Due to the fact that TNFα induces a significantly higher titer of virus than CLBL, viral supernatants were diluted to equal p24 concentrations and then used to infect U373-MAGI-CXCR4_CEM_ cells to analyze viral infectivity. U373-MAGI-CXCR4_CEM_ cells are human glioblastoma cells that have been transduced to constitutively express CD4 and CXCR4. Additionally, they express β-Galactosidase (β-Gal) from an HIV-1 LTR promoter (Tat-inducible expression) and as such, are regularly used to obtain HIV-1 titers [55]. Forty-eight hours post-infection, we analyzed β-Gal activity in infected cell lysates. As shown in Figure 5B, cells infected with virions produced from CLBL-stimulated OM-10.1 cells expressed five times less β-Gal than cells infected with the same amount of virions produced from TNFα-stimulated cells, indicating that virus produced from PI-treated cells exhibit reduced infectivity. Additionally, these results corroborate the data presented in Figure 4. These experiments delineate the ability of PIs to both activate latent virus in a population of cells and inhibit the replication of resulting virus from that population of cells within a treatment duration of only three days. Therefore, it is expected that continuous PI treatment over several rounds of infection would result in an exponential decline in both latent and replicating HIV-1.

## Discussion

This report demonstrates that PIs have the ability to activate latent HIV-1 in three tissue culture model systems and two primary cell model systems, and thereby identifies PIs as a new class of HIV-1 latency antagonists. We have also confirmed that proteasome inhibition in producer cells results in reduced viral titers and the production of virions that exhibit reduced infectivity. Taken together, our results represent a novel proof-of-concept that a single pharmaceutical drug can antagonize both HIV-1 latency and replication simultaneously.

The identification of the proteasome as a potential cellular regulator of the maintenance of HIV-1 latency, from our preliminary genetic screen (unpublished data), was initially surprising considering that proteasome activity is involved in the activation of NFκB, a transcription factor known to potently activate HIV-1 transcription (Reviewed in [56]). However, a review of the literature revealed two gene expression profile studies that supported this finding. One study analyzed the gene expression profile in latently infected ACH-2 cells and found that 15 proteasome genes were upregulated in the latent state prior to viral reactivation [57]. The other study, which analyzed gene expression profiles in productively infected and latently infected cells, found that seven proteasome genes were downregulated in productively infected H9 cells in comparison to uninfected cells [58]. This information, in accordance with our preliminary results, led us to formulate the hypothesis that the proteasome is involved in maintaining HIV-1 latency. Indeed, as shown in Figures 1, 2, and 3, inhibiting the proteasome significantly activated latent viral transcription in OM-10.1 cells as well as latent viral gene expression in all tissue culture and primary cell-based HIV-1 latency models tested. There is one study that seemingly contradicts our findings that PIs activate latent proviral transcription in which it was reported that PIs downregulate HIV-1 LTR-dependent gene expression [52]. However, their results were obtained from experiments in which luciferase expression was analyzed in cells that had been transiently co-transfected with pLTR-LUC and a p65 overexpression plasmid (to induce LUC expression) 32 hours prior to the addition of PIs. In that scenario, luciferase transcription was initiated prior to the addition of PIs and therefore, their results do not reflect a PI-mediated effect on transcriptional initiation. Our studies indicate that PIs induce the initiation of latent proviral transcription, which then results in an upregulation of gene expression. Also, our results were obtained from experiments utilizing fully integrated, latent HIV-1, which is a more relevant system for analyzing the regulation of LTR-driven gene expression.

The variability in the degree of activation of viral transcription and gene expression between all inhibitors and all model systems in the current study could be attributed to differences in toxicities and pharmacokinetics associated with each drug within each model system. Variability might also be affected by the specific model system and mode of reporter gene analysis. For example, late gene products were analyzed in the tissue culture model systems (RLUC and SEAP) while an early gene product was analyzed in the primary cell model systems (GFP). In addition, a degree of variability is expected when comparing results in primary cells obtained from different donors [59,60]. Nevertheless, PIs significantly activated latent virus in all five model systems tested and therefore, the results identify PIs as a new, potent class of HIV-1 latency antagonists. The primary latent cell models in this study were utilized instead of cells directly isolated from HIV-1 infected patients for a specific reason. Generally, when using patient samples for latency activation studies, the addition of allogeneic, MHC mismatched CD4^+^ T cells is required to allow viral outgrowth upon latent viral reactivation [4,61-64]**.** The addition of allogeneic cells themselves might contribute to the activation of latent virus in patient sample systems and therefore, the latently infected primary cell models, described in the results section, were chosen for testing the antagonist activity of PIs.

It is of note that we were able to confirm previous findings [47,48] that PIs do not activate primary human resting CD4^+^ T cells *in vitro* (Figure 3D). This is an important consideration with any potential latency antagonist in order to avoid promoting a “cytokine storm” during treatment. In corroboration, studies have indicated that PIs are not prohibitively toxic *in vivo*, as the PI Velcade is FDA approved for the treatment of multiple myelomas, leukemias, and lymphomas [37,39-42].

There are potential explanations for PI-mediated activation of latent HIV-1. First, there is increasing evidence that the 26S proteasome and/or individual 19S and 20S proteasome subparticles can regulate transcription both proteolytically and non-proteolytically [65]. Interestingly, a study found that the 26S proteasome is associated with the HIV-1 LTR and controls basal transcription proteolytically in the absence of Tat in HeLa-LTR-Luc cells [66,67]. Therefore, it is possible that PIs facilitate the activation of latent HIV-1 by inhibiting the degradation of factors that are involved in promoting viral transcription. For example, it was recently shown that the proteasome partially downregulates the expression of Cyclin T1, a subunit of the P-TEFb complex, in resting memory human CD4^+^ T cells [68]. The P-TEFb complex is essential for HIV-1 transcriptional elongation as a cofactor of Tat (Reviewed in [56]). It is theorized that low levels of Cyclin T1 contribute to the establishment of latency in resting memory CD4^+^ T cells because they are less capable of supporting processive viral transcription [68]. Hence, PIs might promote the activation of latent HIV-1 transcription by stabilizing the Cyclin T1 component of the P-TEFb complex. It will be of interest to explore the mechanism(s) of PI-induced activation of latent HIV-1 in more detail.

Experiments were also performed indicating that PIs inhibit HIV-1 replication by reducing viral output and virion infectivity (Figures 4 and 5). These findings corroborate previous reports. One report found that the PI Lactacystin reduced viral release from cells transfected with HIV-1_NL4-3_ by approximately 3-fold. Moreover, they found that MG-132 decreased the infectivity of virions released from treated, HIV-1_NL4-3_ infected human A3.01 T cells by 50-fold. It should be noted that the concentration of MG-132 used in that study was 100 times higher (40-50 μM) than the concentration used here, which could explain the fact that they observed a much higher-fold reduction in infectivity [51]. Another report found that activated PBMCs (collected from healthy donors) infected with HIV-1_BAL_ in the presence of Velcade and/or MG-132 exhibited an approximate 10-fold reduction in both viral supernatant reverse transcriptase and proviral copy number in target cells [52]. Finally, three genome-wide knock-down screens, performed to identify cellular modulators of HIV-1 replication, identified the proteasome complex as an enriched gene ontology functional group whose downregulation inhibited HIV-1 replication [69].

The bulk of evidence suggests that the APOBEC3 family of cellular viral restriction factors, among which APOBEC3G and 3F (A3G/F) are the most potent, might explain the PI-induced reduction in virion infectivity observed, especially considering our data indicating that PIs exert their effects only when added to virus-producing cells. A3G/F are cytidine deaminases that have been shown to inhibit HIV-1 infectivity by inducing viral genome hypermutation as well as by causing defects in the efficiency of reverse transcription and integration. In order to inhibit viral infectivity, A3G/F must be expressed in virus-producing cells and packaged into nascent virions. From there, they are able to inhibit viral replication in subsequent target cells (Reviewed in [31]). A3G causes viral genome hypermutation during reverse transcription by deaminating cytidine residues in the minus strand of viral DNA, which results in G→A mutations in the plus strand of the viral DNA [70-73]. Additionally, A3G was shown to impair primer tRNA processing during reverse transcription, which not only affects the progression of reverse transcription but also results in the creation of viral DNA ends that are unfit for integration into cellular DNA [74-76]. However, under normal conditions, HIV-1 is protected from the effects of A3G/F by the viral protein Vif, which binds to and targets A3G/F for proteasomal degradation precluding its incorporation into HIV-1 virions [53,77-81].

Numerous studies have shown that PIs increase intracellular levels of A3G in infected cells by inhibiting its degradation even in the presence of Vif [53,77-79,81]. One study found that A3G was incorporated into virions produced from MG-132 treated virus-producing 293T cells and those virions exhibited an approximate 5-fold reduction in infectivity [53]. A3G/F are expressed in primary CD4^+^ T cells and OM-10.1 cells [31], both of which were used to produce virus during treatment with PIs in this study. Therefore, it is a strong possibility that the PI-mediated inhibition of HIV-1 infectivity observed here was in large measure mediated by A3G/F. Explanations for the mechanism through which PIs reduce viral titers might involve the stabilization of TETHERIN, an extracytosolic membrane protein known to inhibit viral release (Reviewed in [30]) or the dysregulation of HIV-1 p6^Gag^, known to promote viral budding and to be regulated via monoubiquitination [51]. It will be interesting to delineate these mechanisms, as well as potential alternative mechanism(s) of PI-mediated inhibition of HIV-1 replication in future studies.

Overall, the data presented in this report validate the concept that effective inhibition of both HIV-1 latency and replication is attainable through the use of a single drug. The importance of this is underlined by evidence of differential antiretroviral drug efficacy within viral pools in secondary lymphoid tissue compartments in patients. For instance, a study observed only mild viral inhibition in the secondary lymphoid tissues of patients on ART regimens made up of two or three reverse transcriptase inhibitors [82]. Also, macrophages have been shown to require the highest therapeutic concentrations of protease inhibitors attainable *in vivo* to inhibit virus production in humans [83]. Moreover, macrophages have been shown to harbor latent HIV-1 in sanctuary tissues, such as the brain [10,17-21], that can be impervious to at least some antiretroviral drugs. For instance, differences in the development of drug resistance between viral isolates from the blood and isolates from the cerebrospinal fluid in patients on HAART indicate that antiretroviral drug penetration into the CNS is not sufficient [22]. Many antiretroviral drugs do not effectively penetrate the BBB [22-28]. The testes represent another potential sanctuary site from which latently infected cells have been isolated [83,84] but in which the blood-testes barrier restricts the entry of some antiretroviral drugs [83,85]. Therefore, tissues that are poorly penetrated by antiretroviral drugs represent potential sites in which re-activated latent virus might infect new cells and re-seed the latent reservoir. In fact, viral replication in patients resulting in more than 50 HIV-1 RNA copies/ml of blood plasma has been shown to decrease the decay rate of the latent reservoir [86,87]. Consequently, in the effort to eliminate latent infection in patients, it is imperative to not only deliver latency antagonists and antiretroviral drugs simultaneously, but to deliver them to the same tissue and cellular compartments. Hence, the development of a single, bifunctional antagonist of both HIV-1 latency and replication is ideal.

Fundamentally, this concept can be applied to the future development of innovative anti-HIV-1 pharmaceuticals capable of clearing latent virus. It is not limited to the use of PIs, as one can imagine employing combinatorial chemistry between different HIV-1 latency activators and replication inhibitors to create novel classes of bifunctional HIV-1 antagonists. However, a PI may very well be a viable option for evaluating the efficacy of an HIV-1 bifunctional antagonist in patients. As previously mentioned, Velcade is already approved by the FDA for the treatment of multiple myeloma and another PI, Marizomib, is currently being evaluated in clinical trials for the treatment of patients with lymphomas, leukemias, and multiple myeloma [37,39]. The most common adverse effects associated with Velcade include gastrointestinal effects, fatigue, thrombocytopenia, and peripheral neuropathy, with the latter two being the most clinically significant [41,42]. However, thrombocytopenia was found to wane between cycles of treatment with Velcade [42] and peripheral neuropathy was found to be effectively managed with dose changes [40]. Perhaps even more promising, early phase I clinical trial results with Marizomib have revealed that at less than half the dose of Velcade, Marizomib is more effective and far less toxic. Importantly, Marizomib is characterized by a very small size that allows it to pass the BBB [37], which could be quite important in purging the latent reservoir in patients. Thus, PIs have exhibited substantial efficacies and manageable toxicities in patients with multiple myeloma and therefore, PIs could be considered feasible candidates for an assessment of the efficacy of HIV-1 bifunctional antagonists in infected patients. Also, PIs target a cellular factor, which significantly increases the genetic threshold for the development of drug resistance.

## Conclusions

In this study, PIs are shown to represent a new class of HIV-1 latency antagonists. Additionally, by confirming their anti-replication activity, it is determined that PIs can act as bifunctional antagonists of HIV-1 latency and replication. Therefore, the findings demonstrate the feasibility of developing effective dual-acting inhibitors of HIV-1. This is a novel concept that can be applied to the development of pioneering anti-HIV-1 pharmaceuticals with the potential to substantially impact the goal of purging HIV-1 from infected individuals.

## Methods

### Plasmid constructs

The construct present in HeLa#14 cells (RLUC/RFP) is an HIV-1_NL4-3_-based construct harboring *Renilla* luciferase (RLUC) in the *env* position and red fluorescence protein (RFP) in the *nef* position [43]. The construct present in 24ST1NLESG cells (SEAP/GFP) is an HIV-1_NL4-3_-based construct harboring secreted alkaline phosphatase (SEAP) in the *env* position and green fluorescence protein (GFP) in the *nef* position [44]. Both the RLUC/RFP and the SEAP/GFP constructs have a 2.5-kb deletion in *pol* and a 1.0-kb deletion in *env* to render the vectors replication-incompetent. Additionally, the *vpu* start codon in both constructs is mutated for robust marker gene expression [43,44]. The *BCL2* expression vector pEB-FLV [46] was a kind gift from Dr. Robert F. Siliciano. The gGnΔ construct is an HIV-1_NL4-3-_based construct that is replication-incompetent. PCR fragments containing the 3′ region of *env*, the complete coding sequence for *Gaussia* luciferase (GLUC), the T2A sequence, the complete coding sequence for enhanced green fluorescence protein (EGFP), and the 5′ region of *nef* were fused together by SOEing PCR. The T2A sequence directs bicistronic expression [88,89]. The fused PCR fragment was inserted into the HIV-1_NL4-3_ construct via *Bam*HI-*Xho*I restriction sites. A 903-bp deletion in *env* was made by inserting a PCR fragment, containing the 3′ region of *vpr* including a mutation in the start codon of *env* (ATG → ACG) and the 5′ region of *env* up to position 6344, into the HIV-1_NL4-3_ construct via *Eco*RI-*Nhe*I restriction sites. The Gn construct is an HIV-1_NL4-3_-based construct that is replication-competent. PCR fragments containing the 3′ region of *env*, the complete coding sequence for EGFP, the T2A sequence, and the 5′ region of *nef* were fused together by SOEing PCR. The T2A sequence directs bicistronic expression [88,89]. The fused PCR fragment was inserted into the HIV-1_NL4-3_ construct via *Bam*H1-*Xho*1 restriction sites.

### Cells and culture media

The following cell lines were obtained through the AIDS Research and Reference Reagent Program, Division of AIDS, NIAID, NIH: OM-10.1 from Dr. Salvatore Butera [32-36], and U373-MAGI-CXCR4_CEM_ from Dr. Michael Emerman [55]. OM-10.1 cells, primary human activated CD4^+^ T cells, primary human resting CD4^+^ T cells, primary human CD4^+^ T_CM_-like cells, and primary human CD4^+^*BCL2*-transduced cells were cultured in RPMI 1640 GlutaMAX, HEPES medium (Life Technologies, Grand Island, NY) supplemented with 10% FBS (Thermo Scientific Hyclone, Logan, UT), 2X MEM Non-essential amino acids solution (Life Technologies, Grand Island, NY), and 100 U/ml penicillin-100 μg/ml streptomycin solution (Life Technologies, Grand Island, NY). HeLa#14 cells were cultured in MEM GlutaMAX medium (Life Technologies, Grand Island, NY) supplemented with 10% FetalClone III Serum (Thermo Scientific Hyclone, Logan, UT), 2X MEM Non-essential amino acids solution, and 100 U/ml penicillin-100 μg/ml streptomycin solution. U373-MAGI-CXCR4_CEM_ cells were cultured in DMEM, high glucose GlutaMAX medium (Life Technologies, Grand Island, NY) supplemented with 10% FBS, 2X MEM Non-essential amino acids solution, 0.2 mg/ml G418 (Sigma-Aldrich, St. Louis, MO), 0.1 mg/ml hygromycin B (Sigma-Aldrich, St. Louis, MO), and 1.0 μg/ml puromycin (Sigma-Aldrich, St. Louis, MO). HeLaT4 cells and HEK293T cells were cultured in DMEM, high glucose GlutaMAX medium supplemented with 10% FetalClone III Serum, 2X MEM Non-essential amino acids solution, and 100 U/ml penicillin-100 μg/ml streptomycin solution.

### Development of latently infected primary human CD4^+^ T cell models: T_CM_-like cells and *BCL2*-transduced cells

Leukocyte enriched blood samples from healthy adult human donors were purchased from the New York Blood Center. To obtain a purified population of peripheral blood mononuclear cells (PBMCs), buffy coats were isolated from the blood by a Ficoll gradient using Histopaque-1077 (Sigma-Aldrich, St. Louis, MO). Also, red blood cells were lysed using ACK lysing buffer (Lonza, Inc., Allendale, NJ). Primary human CD4^+^ T_CM_–like cells were prepared as previously described [45] with the modification that naïve CD4^+^ T cells were separated from PBMCs using the Dynabeads Untouched™ Human CD4^+^ T Cells kit (Life Technologies, Grand Island, NY) according to the manufacturer’s instructions with the additional antibodies mouse IgG anti-human CD25 (BD Biosciences, Franklin Lakes, NJ) and mouse IgG anti-human CD45RO (BD Biosciences, Franklin Lakes, NJ) for the specific removal of activated and memory CD4^+^ T cells, respectively. Primary human *BCL2*-transduced CD4^+^ T cells were prepared as previously described [46] with the modification that cells were activated using the Dynabeads Human T-Activator CD3/CD28 kit (Life Technologies, Grand Island, NY). Vesicular stomatitis virus envelope pseudotyped virions used to establish a latent viral infection in T_CM_–like cells or in *BCL2*-transduced cells were produced in HEK293T cells transfected with the gGnΔ construct and the packaging plasmid pMD.G [90] using polyethyleneimine linear (molecular weight of 25 kDa) (Polysciences Inc., Warrington, PA). Forty-eight hours post-transfection, viral supernatants were collected and concentrated 50X using Retro-Concentin (System Biosciences, Mountain View, CA) according to the manufacturer’s instructions. T_CM_–like cells or *BCL2*-transduced cells were infected via spinoculation as previously described [91] with the modifications that infections were carried out in 24-well plates and in the presence of 8 μg/ml polybrene (Sigma-Aldrich, St. Louis, MO). Infected cells were cultured for seven days to establish a latent infection [45,46].

### Proteasome function assay

OM-10.1 cells were plated in 12-well plates at 1×10^6^ cells per well. Immediately, cells were treated in duplicate with 1 nM, 15 nM, and 30 nM Velcade (concentration dependence) (Selleckchem, Houston, TX), 5 μM clasto-Lactacystin β-lactone (CLBL) (Sigma-Aldrich, St. Louis, MO), 500 nM MG-132 (Cayman Chemicals, Ann Arbor, MI), or 0.25% DMSO or were left untreated (negative controls). Two hours post-treatment, 1×10^4^ cells from each treatment were collected and proteasome function was measured using the Proteasome-Glo Chymotrypsin-Like Cell-Based Assay (Promega, Madison, WI) according to the manufacturer’s instructions. Luminescence was measured on the Turner Biosystems 20/20^n^ Luminometer using default settings.

### PI treatment to analyze activation of latent HIV-1 transcription

For the time course experiment, OM-10.1 cells were plated in 12-well plates at 1×10^6^ cells per well. Immediately, cells were treated with 15 nM Velcade, 2 μM suberoylanilide hydroxamic acid (SAHA) (Selleckchem, Houston, TX) (positive control), or 0.25% DMSO (negative control). At the indicated time points, total RNA was isolated from cells using TRIzol Reagent (Life Technologies, Grand Island, NY) according to the manufacturer’s instructions. RNA samples were then treated with RQ1 DNase (Promega, Madison, WI) according to the manufacturer’s instructions. Reverse transcription PCR was performed to convert RNA to cDNA using the High-Capacity cDNA Reverse Transcription Kit employing random primers (Applied Biosystems (ABI), Foster City, CA) according to the manufacturer’s instructions. The cDNA was then used as a template for quantitative PCR (qPCR) to determine HIV-1_LAV_*nef* RNA expression levels. The qPCR reactions contained Power Sybergreen PCR Master Mix (ABI, Foster City, CA) and one of the following primer sets: the *nef* primer set (Forward 5′-AAGGGAAAGAATGAGACGAGC-3′ and Reverse 5′-GCTACTTGTGATTGCTCCATG-3′), or the GAPDH (reference gene) primer set (Forward 5′-AATCCCATCACCATCTTCCAG-3′ and Reverse 5′-CTTCTCCATGGTGGTGAAGAC-3′). qPCR was performed using the BioRad CFX 96 Real-Time System C1000 Thermal Cycler with the following program settings: 95°C for 10 minutes, followed by 40 cycles of 95°C for 15 seconds, 60°C for 30 seconds, followed by a melt curve. RNA expression was calculated via ΔΔC(t) method with the values normalized to the expression level of GAPDH in each sample.

For the other RNA experiments, OM-10.1 cells were plated in 12-well plates at 1×10^6^ cells per well. Immediately, cells were treated in duplicate with 1 nM, 15 nM, and 30 nM Velcade (concentration dependence), 5 μM CLBL, 500 nM MG-132, 2 μM SAHA (positive control), or 0.25% DMSO (negative control). Seventy-two hours post-treatment, total RNA was isolated, treated with DNase, and reverse transcribed as described above. The cDNA was then used as a template for qPCR to determine HIV-1_LAV_*nef* and *env* RNA expression levels. qPCR was performed as described above with the addition of the *env* primer set (Forward 5′-GCTTTGTTCCTTGGGTTCTTG-3′ and Reverse 5′-ATAATTGTCTGGCCTGTACCG-3′).

### PI treatment to analyze activation of latent HIV-1 gene expression

Twenty-four hours prior to treatment, HeLa#14 cells were seeded in 24-well plates at 5×10^4^ cells per well. Cells were then treated in triplicate with 4.5 μM CLBL, 450 nM MG-132, 7 nM Velcade, 2 μM SAHA (positive control), or 0.25% DMSO (negative control). Forty-eight hours post-treatment, the cells were lysed, protein concentration was measured via standard Bradford assay (Bio-Rad Laboratories, Hercules, CA) and RLUC activity was measured using the *Renilla* luciferase Assay System (Promega, Madison, WI) according to the manufacturer’s instructions. Luminescence was measured on the Turner Biosystems 20/20^n^ Luminometer with a 10 second integration setting.

24ST1NLESG cells were plated in 24-well plates at 5×10^5^ cells per well. Immediately, cells were treated in triplicate with 4.5 μM CLBL, 450 nM MG-132, 7 nM Velcade, 2 μM SAHA (positive control), or 0.25% DMSO (negative control). CLBL and SAHA treated cells were analyzed 48 hours post-treatment while MG-132, Velcade, and DMSO treated cells were analyzed 72 hours post-treatment. The number of live cells in each sample was determined via standard trypan blue exclusion test and quantification using a hemocytometer. SEAP activity in the culture supernatant was measured using the Phospha-Light Secreted Alkaline Phosphatase Reporter Gene Assay System (ABI, Foster City, CA) according to the manufacturer’s instructions. Luminescence was measured on the Turner Biosystems 20/20^n^ Luminometer using default settings.

OM-10.1 cells were plated in 6-well plates at 2.5×10^6^ cells per well. Immediately, cells were treated in duplicate with 5 μM CLBL, 500 nM MG-132, 1 nM, 15 nM, and 30 nM Velcade (concentration dependence), 2 μM SAHA (positive control), or 0.25% DMSO (negative control). Seventy-two hours post-treatment, HIV-1 capsid protein (p24) concentration in the culture supernatant was analyzed via p24 ELISA using the HIV-1 p24 Antigen Capture Kit (AIDS & Cancer Virus Program, NCI-Frederick, MD) according to their instructions. Secondary antibody peroxidase activity was determined via colorimetric analysis using the Coulter Microplate Reader set to read at 450 nm with a reference reading at 650 nm.

T_CM_–like cells and *BCL2*-transduced cells, latently infected with the gGnΔ construct, were plated in 96-well plates at 1×10^5^ cells per well. Immediately, cells were treated in singlicate with 5 μM CLBL, 250 nM MG-132, 10 nM Velcade, Dynabeads Human T-Activator CD3/CD28 beads according to the manufacturer’s instructions in the presence of 30 U/ml IL-2 (positive control), or were left untreated (negative control). All cells were simultaneously treated with 10 μM Raltegravir to prevent the integration of as yet unintegrated viral genomes. Forty-eight hours post-treatment, cells were fixed with a 1% formaldehyde solution for 5 minutes and then GFP mean channel fluorescence values were determined using the BD Biosciences Accuri C6 Flow Cytometer set to count 2×10^3^ cells per sample.

### PI treatment to analyze T cell activation status

Human PBMCs were isolated from healthy donor leukocyte enriched blood samples as described above. CD4^+^ T cells were isolated using the Dynabeads Untouched™ Human CD4^+^ T Cells kit according to the manufacturer’s instructions. The cells were plated in a 96-well plate at 1×10^5^ cells per well and then, in singlicate, left untreated (negative control), treated with 10 nM Velcade, treated with 500 nM MG-132, or activated using the Dynabeads Human T-Activator CD3/CD28 kit according to the manufacturer’s instructions in the presence of 30 U/ml IL-2 (positive control). Forty-eight hours post-treatment, cells were incubated with 20 μl of mouse IgG FITC-conjugated anti-human CD25 antibody (BD Biosciences, Franklin Lakes, NJ) for 30 minutes at 4°C. The percentage of CD25^+^ cells in each of the samples was then determined using the BD Biosciences Accuri C6 Flow Cytometer set to count 2×10^3^ cells per sample.

### PI treatment to analyze antagonism of HIV-1 infectivity

Human PBMCs were isolated from healthy donor leukocyte enriched blood samples as described above. CD4^+^ T cells were isolated using the Dynabeads Untouched™ Human CD4^+^ T Cells kit according to the manufacturer’s instructions. The CD4^+^ T cells were then activated using the Dynabeads Human T-Activator CD3/CD28 kit according to the manufacturer’s instructions in the presence of 30 U/ml IL-2 and were cultured an additional 3 days in the presence of IL-2. The cells were then plated in 24-well plates at 2×10^6^ cells per well. Immediately, 0.5 ml of Gn virus stock was added to each well and the cells were spinoculated as previously described [91] in the presence of 8 μg/ml polybrene. Six hours post-spinoculation, the infections were terminated and either 10 nM Velcade was added to the cultures immediately, or the cultures were left untreated as a positive control. Seventy-two hours post-treatment, viral supernatants were collected and p24 concentrations were measured via p24 ELISA using the HIV-1 p24 Antigen Capture Kit according to their instructions. Secondary antibody peroxidase activity was determined via colorimetric analysis using the Coulter Microplate Reader set to read at 450 nm with a reference reading at 650 nm. HeLaT4 cells were seeded in 24-well plates at 2×10^5^ cells per well 24 hours prior to infection. Viral supernatants were diluted 2.5-fold and then 0.5 ml of diluted supernatants were added to each well in duplicate. As a control for the potential effects arising from residual PI in the inoculums collected from PI-treated CD4^+^ T cells, 10 nM Velcade was added to some HeLaT4 cells as they were infected with viral supernatant collected from untreated CD4^+^ T cells. The HeLaT4 cells were then spinoculated in the presence of polybrene as described above. Infections were terminated 6 hours post-spinoculation. Forty-eight hours post-infection, cells were trypsinized and fixed with a 1% formaldehyde solution for 5 minutes and the percentage of GFP^+^ cells were analyzed using the BD Biosciences Accuri C6 Flow Cytometer set to count 2×10^3^ cells per sample.

### PI treatment to analyze dual antagonism of HIV-1 latency and replication

OM-10.1 cells were treated with either 50 ng/ml TNFα (Sigma-Aldrich, St. Louis, MO), to stimulate the production of positive control viral particles, or with 5 μM CLBL, to stimulate the production of viral particles under the influence of proteasomal inhibition. Seventy-two hours post-treatment, virus-containing supernatants were collected and p24 concentrations were measured via p24 ELISA using the HIV-1 p24 Antigen Capture Kit according to their instructions. Secondary antibody peroxidase activity was determined via colorimetric analysis using the Coulter Microplate Reader set to read at 450 nm with a reference reading at 650 nm. Twenty-four hours prior to infection, U373-MAGI-CXCR4_CEM_ cells were seeded in 12-well plates at 1×10^5^ cells per well. Viral supernatants were diluted to a p24 concentration of 200 ng/ml and then 300 μl of the diluted viral supernatants were used to infect U373-MAGI-CXCR4_CEM_ cells in duplicate in the presence of 8 μg/ml polybrene at 37°C. Infections were terminated within 5 hours. Forty-eight hours post-infection, cells were lysed, protein concentration was measured via standard Bradford assay and β-galactosidase activity was measured using the β-Galactosidase Enzyme Assay System with Reporter Lysis Buffer (Promega, Madison, WI) according to the manufacturer’s instructions. Colorimetric analysis was performed using the Nanodrop 2000 Spectrophotometer set to read at 420 nm.

## Abbreviations

AIDS: Acquired immunodeficiency syndrome; A3G/F: APOBEC3G/APOBEC3F; APOBEC3: Apolipoprotein B mRNA-editing, enzyme-catalytic, polypeptide-like 3; ART: Antiretroviral therapy; β-gal: Beta-galactosidase; BBB: Blood–brain barrier; BCL2: B-cell lymphoma 2; cDNA: Complementary deoxyribonucleic acid; CLBL: Clasto-Lactacystin beta-lactone; CNS: Central nervous system; CXCR4: Chemokine (C-X-C motif) receptor 4; DMSO: Dimethyl sulfoxide; DNA: Deoxyribonucleic acid; EGFP: Enhanced green fluorescence protein; ELISA: Enzyme-linked immunosorbent assay; Env: Envelope; FBS: Fetal bovine serum; FDA: Food and drug administration; FITC: Fluorescein isothiocyanate; GAPDH: Glyceraldehyde 3-phosphate dehydrogenase; GFP: Green fluorescence protein; GLUC: *Gaussia* luciferase; HAART: Highly active antiretroviral therapy; HIV-1: Human immunodeficiency virus type 1; IC50: Half-maximal inhibitory concentration; IgG: Immunoglobulin G; LTR: Long terminal repeat; LUC: Luciferase; MCF: Mean channel fluorescence; MHC: Major histocompatibility complex; NCI: National cancer institute; Nef: Negative factor; NIAID: National institute of allergy and infectious diseases; NIH: National institutes of health; NFκB: Nuclear factor kappa-light-chain-enhancer of activated B cells; P-TEFb: Positive transcription elongation factor b; p24: HIV-1 capsid protein; PBMC: Peripheral blood mononuclear cell; PCR: Polymerase chain reaction; PI: Proteasome inhibitor; Pol: Polymerase; qPCR: Quantitative polymerase chain reaction; Rev: Regulator of expression of virion proteins; RFP: Red fluorescence protein; RLU: Relative light unit; RLUC: *Renilla* luciferase; RNA: Ribonucleic acid; SAHA: Suberoylanilide hydroxamic acid; SEAP: Secreted alkaline phosphatase; SEM: Standard error of the mean; SOEing PCR: Synthesis by overlap extension polymerase chain reaction; Tat: Trans-activator of transcription; TNFα: Tumor necrosis factor alpha; tRNA: Transfer ribonucleic acid; Vif: Virion infectivity factor; Vpr: Viral protein R; Vpu: Viral protein U.

## Competing interests

The author(s) declare that they have no competing interests.

## Authors’ contributions

LKM designed and performed the majority of the experiments, analyzed the data, and wrote the paper. YK designed and performed experiments involving primary human CD4^+^ T cells. JPD envisioned the overall concept of the study and participated in the drafting of the manuscript. CCC, TAR, and YR, contributed to the conception and design of the study. All authors read and approved this manuscript.

## Authors’ information

LKM and TAR are graduate students in the Graduate School of Biomedical Sciences at Rutgers University-Robert Wood Johnson Medical School. YK is a research teaching specialist IV in the department of Pharmacology at Rutgers University-Robert Wood Johnson Medical School. CCC is an adjunct assistant professor in the department of Pharmacology at Rutgers University-Robert Wood Johnson Medical School. YR and JPD are professors in the department of Pharmacology at Rutgers University-Robert Wood Johnson Medical School.
